# Case report: Composite mantle cell lymphoma and classical Hodgkin lymphoma

**DOI:** 10.3389/pore.2023.1611051

**Published:** 2023-03-17

**Authors:** Hongyu Wang, Liqun Yang, Qiuyao Li, Haiyun Song, Hong Ji

**Affiliations:** ^1^ Department of Pathology, Qilu Hospital (Qingdao), Cheeloo College of Medicine, Shandong University, Qingdao, China; ^2^ Huayin Health Hematopathology Comprehensive Diagnostic Center, Chengdu Huayin Medical Laboratory Center, Chengdu, China

**Keywords:** next-generation sequencing, classical Hodgkin lymphoma, mantle cell lymphoma, composite lymphoma, histogenesis

## Abstract

Composite mantle cell lymphoma and classical Hodgkin lymphoma is very rare and the actual origin of it is still unclear. Here we reported a new case of composite mantle cell lymphoma and classical Hodgkin lymphoma and analyzed its molecular changes. Eight mutations were identified in its Hodgkin component through next-generation sequencing. In addition, we reviewed the published cases of composite mantle cell lymphoma and classical Hodgkin lymphoma and summarized the molecular changes of reported cases as well as the current case to explore the possible pathway of histogenesis.

## Introduction

Composite lymphoma (CL) is rare, and its incidence is approximately 1.0%–4.7% of all lymphomas. CL is defined as the concurrent or sequential occurrence of two or more distinct types of lymphoma in the same anatomical location or tissue (1). CL can consist of two non-Hodgkin lymphomas (NHL) or a Hodgkin lymphoma (HL) and an NHL (1). The combination of mantle cell lymphoma (MCL) and classical HL (cHL) is extremely rare, and only 10 cases have been reported in the literature since its first description in 2003 by Caleo et al. (2–10). Here we report another case of CL that presented as a composite of MCL and cHL, and we analyzed their molecular characteristics through next-generation sequencing (NGS). In addition, we reviewed published articles and summarized reported cases to explore the possible pathway of histogenesis.

## Case presentation

A 70-year-old male presented with a 3-month history of a right axillary mass without pain, fever, or tiredness. The right axillary mass gradually enlarged, and 1 month later, a new mass developed in the left retroauricular region. The patient received anti-infective therapy in a local hospital, but his condition did not improve. He subsequently visited our hospital on 31 May 2021. Ultrasonography revealed multiple enlarged lymph nodes in the bilateral axillary and inguinal regions. The size of the largest lymph node was 70.3 × 54.5 mm. The patient refused to undergo lymph node biopsy and opted for an anti-infective medicine. However, the lymph nodes continued to enlarge. The patient was admitted to our hospital again on 20 July 2021. A physical examination revealed movable masses with a tough texture in the left retroauricular region and right and left axillae; the largest measurements was 7 × 5 cm. Full blood count showed a slightly increase in white blood cell count (11.26×10^9^/L, normal range: 3.5–9.5×10^9^/L) and lymphocyte count (3.41×10^9^/L, normal range: 1.1–3.2×10^9^/L). The lactic dehydrogenase (LDH) level was normal (226U/L, normal range: 91–245U/L).

Right axillary lymph node biopsy was performed on 23 July 2021. Macroscopically, the mass measured 8.0 × 8.0 × 5.5 cm, with a tough texture appearing as a gray-white to red color on the cur surface.

**FIGURE 1:**
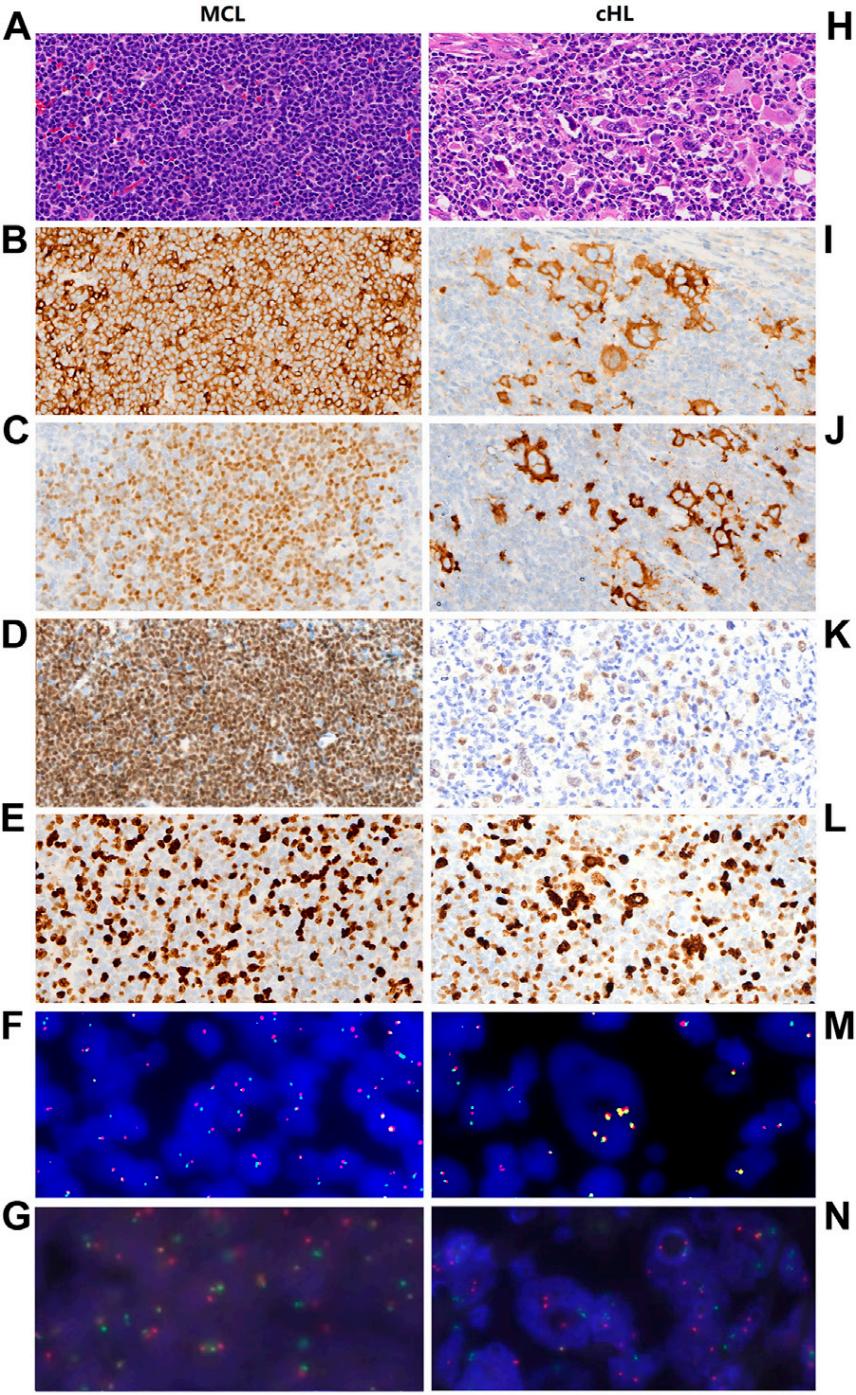
Pathological features and FISH analysis of MCL and cHL components, respectively. Hematoxylin and eosin (HE) staining **(A)** shows that MCL is composed of monomorphic small lymphocytes which resembling centrocytes, these cells show positive immunohistochemistry staining for CD5 **(B)**, CyclinD1 **(C)**, and PAX-5 **(D)**. The proliferation rate of Ki-67 is approximately 40% **(E)**. FISH analysis shows CCND1 gene break **(F)** as well as IgH/CCND1 gene fusion **(G)** in MCL. In cHL region, HE staining **(H)** demonstrates that cHL component consists of mixed inflammatory cells and large HRS cells. HRS cells show positive immunohistochemistry staining for CD30 **(I)**, CD15 **(J)**, PAX-5 **(K)** and the proliferation rate of Ki-67 is approximately 70% **(L)**. In HRS cells, FISH analysis dose not demonstrate CCND1 gene break **(M)** and IgH/CCND1 gene fusion **(N)**.

Microscopically, the lymph node comprised two components. One component consisted of monomorphic small lymphocytes with a vague nodular pattern (Figure 1A). These lymphocytes resembled centrocytes with slightly irregular nuclei and inconspicuous nucleoli. They weakly expressed CD5 (Figure 1B) and were positive for CyclinD1 (Figure 1C), PAX-5 (Figure 1D), CD20, OCT-2, and BOB-1. The proliferation rate of Ki-67 was approximately 40% (Figure 1E). The other component of the lymph node showed scattered large mononuclear Hodgkin cells and binuclear or multinuclear Reed-Sternberg cells in a background of mixed inflammatory cells (Figure 1H). These large cells had enlarged irregular nuclei and prominent eosinophilic nucleoli. Immunochemistry revealed positive staining of Hodgkin and Reed-Sternberg (HRS) cells for CD30, CD15, and PAX 5 (Figures 1I–K); decreased expression of CD20, OCT-2, and BOB-1; and negative expression of CD5 and CyclinD1. The proliferation rate of Ki-67 in the HRS cells was approximately 70% (Figure 1L). The background of the HRS cells was rich in lymphocytes and epithelioid histiocytes with a small number of scattered eosinophils, plasmocytes, and neutrophils. Immunochemical staining showed that CD4-positive T cells were significantly more than CD8-positive T cells, and CD3-positive T cell rosettes were observed around the HRS cells. A vague transition was observed between the two lymph node components. In the borderline section, CyclinD1-positive cells and HRS cells were mixed. All the immunohistochemical findings are summarized in Table 1.

**TABLE 1:** Immunohistochemistry analyzed in MCL and HRS cells of CMLHL.

Antibody	Clone	MCL	CHL	Antibody	Clone	MCL	CHL
CD45	2B11	+	−	MUM-1	EP190	−	Weak +
CD20	L26	+	Weak +	C-myc	EP121	−	−
CD79α	EP82	+	Weak +	CD10	56c6	−	−
PAX-5	SP34	+	Weak +	TIA-1	2G9A10F5	−	−
OCT-2	MRQ-2	+	Weak +	CD3	SP7	−	−
BOB-1	SP92	+	Weak +	CD4	EP204	−	−
SOX-11	MRQ-58	−	−	CD8	SP16	−	−
CD5	MX052	+	−	ALK	5A4	−	−
CyclinD1	SA38-08	+	−	EMA	E29	−	−
CD30	Ber-H2	−	+	CKpan	AE1/AE3	−	−
CD15	Carb-3	−	+	P53	DO-7	+, 15%	+, 60%
CD21	EP64	FDC +	FDC +	Ki-67	MIB-1	+, 40%	+, 70%

FDC, follicular dendritic cell.

*In-situ* hybridization for Epstein-Barr virus encoded RNA (EBER) showed no evidence of Epstein-Barr virus (EBV) infection in the tumor. Subsequently, fluorescence *in situ* hybridization (FISH) was performed to examine MCL cells and HRS cells for the typical t (11; 14) MCL translocation. CCND1 gene break was confirmed in the MCL cells (Figure 1F) but not in the cHL cells (Figure 1M) using FISH gene break detection (Abbott, Des Plaines, United States). Moreover, immunoglobulin heavy chain (IGH)/CCND1 fusion translocation was observed in the MCL cells (Figure 1G) but not in the cHL cells (Abbott, Des Plaines, United States) (Figure 1N).

Polymerase chain reaction (PCR) amplifications were performed using BIOMED-2 primers to amplify the immunoglobulin chain (IG) and T-cell-receptor (TCR) genes. Visualization was performed using the GeneMapper software version 4.1. A paraffin block that contained the MCL and cHL components was chosen and was dissected to obtain a pure MCL component and relatively pure cHL component. PCR amplification was performed separately for the two components and it demonstrated clonal rearrangement of IG genes in the MCL and cHL components; a unique clonal IGH (DH-JH, about 200 bp), which was distinct from that found in the MCL component, was found in the cHL component. No rearrangement of TCR genes was detected in the MCL and cHL components

Finally, the patient was diagnosed with composite mantle cell lymphoma and classical Hodgkin lymphoma (CMLHL).

Subsequently, we dissected a paraffin block of CMLHL and removed the borderline section between the MCL and cHL components. Therefore, molecular characteristics were analyzed separately in pure MCL and relatively pure cHL sections using NGS. NGS analysis was performed using haematopoietic- and lymphoid-specific panels covering coding sequences (CDSs) of 318 hematological disease genes relevant to lymphohematopoietic disease using Illumian NextSeq 550 with a mean sequencing depth of 1000x. Data were analyzed using in-house bioinformatics pipeline (KingMed Diagnostics, Chengdu, China) which has a rigorous quality control system. According to the NGS results, only eight mutations, which were classified as uncertain clinical significance (Tier III) were detected in the cHL region, and none was observed in the MCL region. The eight mutations were in FAT1 (c.12616A > G, p. I4206V), ARID1B (c.3562G > A, p. V1188I), PRKDC (c.2449G > C, p. A817P), BIRC3 (c.1471C > T, p. Q491*), KMT2D (c.13780dupG/c.6137dupT, p. A4594Gfs*12/p.M2046Ifs*11), PML (c.1702A > G, p. T568A), CYLD (c.1454A > T, p. Y485F), and BRIP1 (c.2545T > C, p. F849L). Detailed information about the NGS results is presented in Table 2.

**TABLE 2:** The result of NGS in MCL and cHL areas.

	Mutated gene	Transcript ID	Mutated site	Nucleic change	Amino acid change	VAF (%)
MCL	None					
cHL	FAT1	NM_005245	exon25	c.12616A>G	p.I4206V	48.2
cHL	ARID1B	NM_001371656	exon13	c.3562G>A	p.V1188I	47.8
cHL	PRKDC	NM_006904	exon22	c.2449G>C	p.A817P	39.9
cHL	BIRC3	NM_001165	exon7	c.1471C>T	p.Q491*	33.9
cHL	KMT2D	NM_003482/NM_003482	exon41/exon29	c.13780dupG/c.6137dupT	p.A4594Gfs*12/p.M2046Ifs*11	7.7/31.0
cHL	PML	NM_033250	exon7	c.1702A>G	p.T568A	44.2
cHL	CYLD	NM_015247	exon10	c.1454A>T	p.Y485F	47.3
cHL	BRIP1	NM_032043	exon18	c.2545T>C	p.F849L	53.1

VAF, variant allele fraction.

Bone marrow biopsy revealed only MCL infiltration of the bone marrow, but no evidence of cHL infiltration was found. Positron emission tomography-computed tomography highlighted fluorodeoxyglucose avid lymph nodes in the head and neck regions as well as in multiple regions including the spleen. Therefore, the patient was classified as stage IV.

The patient refused chemotherapy and requested to be discharged from the hospital. In October 2022, the patient was still living with the disease.

## Discussion

CL is defined as two or more distinct types of lymphoma occurring concurrently or sequentially in the same anatomical location or tissue (1). Generally, MCL is derived from pre-germinal center B (GCB) cells, and cHL is derived from GCB cells. Considering the different cell origins of MCL and cHL, it is not surprising that a CL composed of MCL and cHL is rare. Until now, ten cases (nine English cases and one Chinese case) have been reported (2–10), and the clinicopathological features of these 11 CMLHL cases including the current case are summarized in Supplementary Table S1.

Of the 11 cases, nine are male and two are female, with a median age of 67.4 years (range, 42–89 years). Most cases presented as lymphadenopathy, especially in the neck or axilla, splenomegaly, and a tonsillar mass. In seven of the 11 cases, the two components of CMLHL were observed simultaneously, while all the other four cases had a previous history of MCL before the cHL component developed after a period. Information about the clinical stages of only two cases have been reported, and both cases were classified as stage IV. Follow-up results were reported for six cases; two showed complete resolution (7, 9), two were alive with the disease (10), and the other two patients had died independent of CMLHL (4, 5).

In eight cases of CMLHL, *in-situ* hybridization for EBER or immunohistochemistry for the EBV encoded latent membrane protein 1 (LMP1) was performed. The EBV infected the HRS cells but not the MCL cells in five of the eight cases. This phenomenon may suggest a possible role of EBV in promoting the occurrence of cHL but not MCL. Interestingly, the case reported by Tinguely et al. (3) showed that only part of the HRS cells was infected by the EBV. Somatic mutation analysis revealed a higher mutation rate in EBV-positive HRS cells compared with EBV-negative HRS cells, which may further prove the important role of EBV in the development of cHL.

Until now, most of the studies that reported CMLHL cases have investigated molecular changes and discovered different clonal relationships between the MCL and cHL components. Thus, several different hypotheses about the origin of each component of CMLHL as well as the clonal relationship between them have been reported, but no unified conclusion has been made. In this study, we collated the molecular changes in the current and reported CMLHL cases.

According to previous reports, six of the seven simultaneous CMLHL cases demonstrated t (11; 14) translocation; four of the six cases (cases 1, 2, 6, and 11) demonstrated t (11; 14) translocation in the MCL cells but not in the HRS cells (2, 6). In case 7, which was a combination of Blastoid MCL and cHL, t (11; 14) translocation was demonstrated in the two components (7). In case 4, the existence of t (11; 14) in the tumor was described, but the difference between the two components was not reported (4). In three of the other four CMLHL cases with a previous history of MCL, two (cases 3 and 5) had t (11; 14) in both the MCL and HRS cells as identified using FISH (3, 5, 13). Although case 9 (1/3) also had t (11; 14), the components involved were not reported in this study (9). Case 10 (1/4) was first diagnosed as MCL, and 1 year later, Blastoid MCL and cHL was found in the same anatomical location. In this case, all the three components revealed t (11; 14) (10).

According to the t (11; 14) translocation analysis, components of CMLHL that were observed simultaneously were likely to have different t (11; 14) results. This may imply that in most CMLHL cases in which the components are observed simultaneously, the cHL components may be clonally unrelated to the MCL components. Furthermore, Caleo et al. (2) detected immunoglobulin heavy chain variable region (IgVH) gene rearrangement in two cases and reported different monoclonal amplification peaks in the MCL and HRS cells, which further confirmed that the two CMLHL components developed from different B cell origins. The case reported by Ciara et al. (7) was an exception because the characteristic t (11; 14) of MCL was also identified in the HRS cells. The result suggested that a clonal relationship existed between Blastoid MCL and cHL in their case. We suspected that the patient may have had a previously undiscovered MCL, which later progressed to Blastoid MCL and cHL. Thus, both secondary components may have inherited t (11; 14) from the previous MCL.

Cases 3, 5, and 10 had a history of MCL, and each component of them showed similar molecular changes in t (11; 14). This phenomenon suggests that in CMLHL secondary to MCL, a clonal relationship may exist between all the CHLML components, and all the CHLML components probably originated from a common precursor cell. We speculated that MCL stimulated by chemotherapy drugs may be transformed into cHL and subsequently lead to CMLHL; thus, the cHL component of CMLHL will inherit t (11; 14) from a previous MCL. Somatic mutation was further detected in cases 3, 5, and 10. Somatic hypermutation (SHM) was discovered in the cHL components but not in the MCL component in case 3 (3, 11). However, in cases 5 and 10 (5, 10), a similar SHM rate was not only found in the cHL component, but also in the MCL component and Blastoid MCL, which suggests that all components including MCL originated from a precursor B cell that was derived from a (post)-GCB cell. Although the derivation of MCL from (post)-GCB cell is unusual, the MCL component in CMLHL with a clonal relationship with other components seemed more likely to have a (post)-GCB cell precursor. Considering the same (post)-germinal center origin, we speculated that MCL derived from a (post)-GCB cell may have a higher probability of being stimulated by drugs and transformed to cHL. Due to the limitation in the number of cases, this speculation still needs to be further confirmed with future studies.

Until now, there have been only few reports of the molecular analysis of CMLHL, and the actual origin of CMLHL is still unclear. There may be three possible pathways responsible for the genesis of CMLHL. Firstly, as shown in the analysis by Caleo et al. (2), CMLHL components have entirely different molecular characteristics. This suggests that they were derived from different precursor cells and together comprise CMLHL as independent lymphomas. Secondly, considering that similar molecular changes of t (11; 14) were found in both the MCL and HRS cells, these two components may have developed from a common precursor cell that contained t (11; 14). Subsequently, the common precursor cell evolves into MCL or cHL by undergoing different transforming events. Thirdly, CMLHL components still originate from a common precursor cell, but the precursor cell may first evolve into MCL, and subsequently, the MCL may undergo further transforming events such as transformation due to drug therapy, and HRS cells will develop directly from MCL. These speculations need to be confirmed by more detailed case information as well as further studies. In the current case, the MCL and cHL components demonstrated different molecular changes: t (11; 14) was only found in the MCL cells, a unique clonal IGH and CDS mutations were detected in only the cHL region. Different molecular changes of MCL and cHL in the current case suggest that they may have derived from different precursor cells and developed into CMLHL through the first pathway.

In our current case, we analyzed the CDSs of haematopoietic- and lymphoid-specific genes in CMLHL using NGS. Eight mutations with uncertain clinical significance (Tier III) were detected in only the cHL region and no mutation was detected in MCL region. A study found that PRKDC had a high mutation rate in several tumors, such as colorectal cancer, gastric cancer, and endometrial cancer, and played an important role in the genesis, development, invasion, and metastasis of tumors through various mechanisms (12). However, PRKDC mutation is rare in lymphoma. In a previous study, PRKDC mutation with uncertain clinical significance was detected in cHL (13). This result is similar to our results, and PRKDC might be a novel putative predisposition gene for cHL. According to the literature, ARID1B (14), PML5 (15), and CYLD (16) has a tumor suppressor function. Mutation-induced loss of function in any of them may have favored to the genesis and development of the cHL component in the current case. According to previous studies, some mutations detected in the current case are associated with a poor prognosis in hematological tumors. For example, BIRC3 mutation is associated with worse outcomes in chronic lymphocytic leukemia (CLL) and acute lymphoblastic leukemia (ALL) (18), KMT2D mutation was considered to be associated with poor prognosis in extranodal NK/T-cell lymphoma and MCL (19, 20), and FAT1 mutation was associated with a poor prognosis and high tumor recurrence rate in patients with T-cell ALL (21). However, the prognostic effect of the three mutations in cHL has not been reported, and their influence on the prognosis of CMLHL is uncertain. BRIP1 mutation is associated with an increased risk of epithelial ovarian cancer, and might be targeted by PARP inhibitors such as olaparib (22). BRIP1 mutation in cHL have been reported in only one study (22). In our current case, limited to experimental conditions, we did not do the microdissection. Thus, we only can confirm that the eight mutations are present in the CHL region. The following three speculations have been made about the component of the cHL region from which the eight mutations originated: 1) All mutations originate from the HRS cells, 2) some mutations may present in the inflammatory cells of the cHL component, and 3) the mutations we detected might present in the subclone of the MCL cells, which is mixed in the cHL region. However, these speculations need to be verified with further studies.

HRS-like cells, which resemble HRS cells in morphology and immunophenotype, can be occasionally reported in B-cell or T cell lymphomas. Thus, it is challenging to differentiate HRS from HRS-like cells when diagnosing a CMLHL. The following reported criteria (23, 24) could help in the recognition of HRS-like cells: 1) the HRS-like cells usually present as scattered single cells or small cluster distributions among NHL and 2) absence of a typical cHL background microenvironment (fibrosis and/or stromal inflammatory reaction). In the current case, the MCL and cHL components were roughly separated. In the cHL component, the background contained collagen as well as mixed inflammatory cells, and the HRS cells showed positive staining of CD15 and CD30, weak positive staining of PAX-5, decreased expression of CD20, OCT-2 and BOB-1, and negative expression of CD45, which are the classic immunophenotype of cHL tumor cells. CCND1 gene break and IGH/CCND1 fusion translocation were not observed in the cHL components. The unique clonal IGH and CDS mutations, which were detected in only the cHL region, further helped us to confirm that the cHL components is an independent tumor instead of a transformed section from MCL, and the diagnose of CMLHL can be confirmed.

In conclusion, we reported a new case of CMLHL and analyzed its molecular changes by FISH, TCR and IG rearrangement, and NGS. We reviewed reported cases and summarized them, with the current case included, to explore the possible pathway of histogenesis. In our current case, we speculated that its MCL and cHL components may have been derived from different precursor cells.

## Data Availability

The original contributions presented in the study are included in the article/Supplementary Material, further inquiries can be directed to the corresponding author.
