# Supplementary material for: Case report: Composite mantle cell lymphoma and classical Hodgkin lymphoma
Source: Pathol Oncol Res. 2023 Mar 17;29:1611051. doi: 10.3389/pore.2023.1611051 (PMC10064289; doi:10.3389/pore.2023.1611051)
Supplement: Supplementary file 1 [file Table1.docx]

Supplementary Table 1. Clinicopathologic characteristics of 11 cases of CMLHL

| case | Author | Gender/Age (year) | Time of onset | Physical examination | Biopsy site | Composition of tumor | EBV | t(11;14) | Other molecular analysis | Stage | therapy | prognosis |
| --- | --- | --- | --- | --- | --- | --- | --- | --- | --- | --- | --- | --- |
| 1 | Caleo A (2003) | Male/61 | At one time | splenomegaly | Spleen | MCL  cHL | (-)  HRS cell (+) | MCL: (+)  HRS cell: (-) | IgV_H_ clonal peaks:  MCL: 134 bp  HRS cell: 134 bp | NA | NA | NA |
| 2 | Caleo A (2003) | Male/66 | At one time | left tonsillar mass, cervical left lymphadenopathy, eyelid nodular mass | cervical left lymphadenopathy and eyelid mass | MCL  cHL | (-)  HRS cell (+) | MCL: (+)  HRS cell: (-) | IgV_H_ clonal peaks:  MCL: 131 bp  HRS cell: 129,146 bp | NA | NA | NA |
| 3 | Tinguely M (2003) | Male/42 | One after another | NA | Abdominal lymph node | MCL (1984)  MCL + cHL (1987) | (-)  Part of HRS cells (+) | No major t(11;14)  MCL: (+)  cHL: (+) | Sequence analysis:  MCL: IgV_H_: 0 mutation; IgVλ: 0 mutation  HRS EBV-: IgV_H_: 2 mutations; IgV_λ_: 3 mutations  HRS EBV+: IgV_H_: 4 mutations; IgV_λ_: 4 mutations | NA | MCL（1984）：KNOSPE + COP  MCL+cHL（1987）：NA | NA |
| 4 | Hayes SJ (2006) | Female/69 | At one time | right cervical and bilateral axillary  lymphadenopathy | axillary lymph node | MCL  cHL | (-)  (-) | (+) | NA | NA | CHOP  fludarabine/cyclophosphamide | Died ^1^ (11 months) |
| 5 | Stefanie S (2014） | Male/70 | One after another | Splenomegaly (2008)  abdominal, cervical and axillary lymphadenopathy (2010) | peripheral blood smear (2008)  lymph node and bone marrow biopsy (2010) | MCL (2008)  MCL + cHL (2010) | NA | NA  MCL: (+)  cHL: (+) | NA  PCR: MCL,cHL both showed VH3-66/DH1-26/JH4 gene rearrangement  Sequence analysis: MCL, cHL both showed somatically mutation rate of 2.8% | NA | MCL (2008): follow up  MCL + cHL (2010): vinblastine-adriamycin-dacarbazine alternating, R-CHOP. | Died ^2^ (time: NA) |
| 6 | Giua R (2015) | Female/89 | At one time | multiple mediastinal, bilateral axillary lymphadenopathy | axillary lymph node | MCL  cHL | NA | MCL: (+)  HRS cell: (-) | NA | NA | NA | NA |
| 7 | Ciara M (2017) | Male/78 | At one time | generalized  lymphadenopathy and splenomegaly. | Posterior triangle of the neck lymph node | Blastoid MCL  cHL | (-)  HRS cell (+) | Blastoid ML: (+)  HRS cell: (+) | PCR in MCL:  IgV_H_ : VFR1-J, VFR2-J, VFR3-J; 334bp, 263, 120, and135 bp  IgVκ:V-J and V/JC intron-kde; 151, 283, and 279 bp  IgVλ: V-J; 142 bp | NA | R-CHOP like therapy, rituximab-cytosine-arabinoside, high-dose methotrexate and folinic acid | CR (time: NA) |
| 8 | Sharma S (2019) | Male/62 | At one time | enlarged right tonsil, right cervical lymphadenopathy | Right‑side tonsil | MCL  cHL | (-)  Large cell (+) | NA | NA | NA | NA | NA |
| 9 | L Guo (2019) | Male/67 | One after another | Right inguinal and submaxillary lymphadenopathy | Right inguinal lymph node (2014)  Right submaxillary lymph node (2015) | MCL (2014)  MCL + cHL (2015) | (-)  (-) | NA  (+) | NA  PCR: IgV_H_ FR1, IgH FR2 and Igκ Vk-Jk monoclonal rearrangement | NA | MCL (2014): Chinese traditional medicine  MCL + cHL (2015): bortezomib + CHOP | CR (30 months) |
| 10 | Hammad T (2019) | Male/70 | One after another | NA | NA (2018)  Left inguinal and bilateral hilar lymph node (2019)  Left acetabulum (2019) | MCL (2018)  cHL(2019)  Blastoid MCL (2019) | NA | MCL: (+)  cHL: (+)  Blastoid MCL: (+) | NGS:  MCL, cHL and blastoid MCL showed the same IgV_H_ sequence as well as a same mutation rate of 3.5%  MCL: 6 mutations;  cHL: 6 mutations  Blastoid MCL: 12 mutations | Stage Ⅳ MCL (2018)  Stage Ⅳ cHL (2019) | MCL (2018): R-CHOP and lenalidomide, R-HiDAC, lenalidomide  cHL (2019): BV-AVD  blastiod MCL (2019): radiation therapy, rituximab and ibrutinib. | AWD (time: NA) |
| 11  (Current case) | —— | Male/70 | At one time | Right axillary  lymphadenopathy | Right axillary lymph node | MCL  cHL | (-)  (-) | MCL: (+)  HRS cell: (-) | TCR:  MCL, cHL: no arrangement  BCR:  MCL:FR1-JH, FR2-JH and Vk-Jk, about 315bp, 250bp and 282bp  cHL:FR1-JH, FR2-JH, DH-JH and Vk-Jk, about 315bp, 250bp, 200bp and 282bp  NGS:  MCL: 0 mutation  cHL: 8 mutations | Stage Ⅳ CMLHL | NA | AWD (17 months) |

NA: non-available. CR: complete remission. AWD: alive with disease. 1: Died of pneumonia. 2: Died of intestinal obstruction, bacterial sepsis, pneumonia and respiratory failure.
